# Appropriate dose of dexmedetomidine for the prevention of emergence agitation after desflurane anesthesia for tonsillectomy or adenoidectomy in children: up and down sequential allocation

**DOI:** 10.1186/s12871-015-0059-z

**Published:** 2015-05-27

**Authors:** Hee-Soo Kim, Hyo-Jin Byon, Jong-Eun Kim, Yong-Hee Park, Ji-Hyun Lee, Jin-Tae Kim

**Affiliations:** 1 Department of Anesthesiology and Pain Medicine, College of Medicine, Seoul National University, 101 daehang-ro, jongno-gu, Seoul, 110-744 Republic of Korea; 2 Department of Anesthesiology and Pain Medicine, Anesthesia and Pain Research Institute, Yonsei University College of Medicine, 50-1, Yonsei-ro, Seodaemun-gu, 120-752 Seoul, Republic of Korea; 3 Department of Anesthesiology and Pain Medicine, College of Medicine, Inha University, 7-206, 3-ga, Sinheung-dong, Jung-gu, Incheon, 400-711 Republic of Korea; 4 Department of Anesthesiology and Pain Medicine, Chung-Ang University Hospital, 102, Heukseok-ro, Dongjak-gu, 156-755 Seoul, Republic of Korea

**Keywords:** Children, Desflurane, Dexmedetomidine, Emergence agitation

## Abstract

**Background:**

Dexmedetomidine can be used for the prevention of emergence agitation (EA) in children. However, an inadequate dose of dexmedetomidine can induce prolonged sedation and cardiovascular complications. The aim of this study was to evaluate the effective dose of dexmedetomidine for the prevention of EA after desflurane anesthesia for patients undergoing a tonsillectomy or adenoidectomy.

**Methods:**

We enrolled 21 unpremedicated children, between 2 and 12 years, undergoing either a tonsillectomy or an adenoidectomy. General anesthesia was induced using sevoflurane and oxygen, and dexmedetomidine was administered before surgery. Anesthesia was maintained using desflurane resulting in a BIS range of 40–60. In the postanesthetic care unit (PACU), EA (agitation measured at level 4 or more at least once) was assessed on arrival in the PACU,15 min later, and 30 min later. The dose of dexmedetomidine for consecutive patients was determined by the response of the previous patient, using an increment or decrement of 0.1 μg/kg.

**Results:**

The 50 % effective dose of dexmedetomidine for prevention of EA was 0.25 μg/kg (95 % confidence limits, 0.17–0.33 μg/kg), and the 95 % effective dose was 0.38 μg/kg (95 % confidence limits, 0.29–0.39 μg/kg).

**Conclusions:**

For prevention of EA after desflurane anesthesia for 50 % and 95 % of children undergoing tonsillectomies or adenoidectomies, 0.25 μg/kg or 0.38 μg/kg of dexmedetomidine is suggested. Further study is needed to validate the suggested dose of dexmedetomidine to prevent the EA that was identified in the present study.

**Trial registration:**

Clinical Research Information Service KCT0000126.

## Background

Emergence agitation (EA) is one of the most common complications of sevoflurane and desflurane anesthesia in children and requires proper management. The incidence of EA varies in the literature from 2–80 % depending on the anesthetic technique and scoring scale [1–3]. There were many trials conducted to manage or prevent EA in children. Dexmedetomidine, a selective α_2_ agonist, has recently been used with some success as a treatment for the prevention of EA [4]. Various doses (0.15–1.0 μg/kg) of dexmedetomidine have been reported to prevent EA in children undergoing sevoflurane anesthesia [5–10]. Due to respiratory irritation, desflurane is a less popular volatile agent for children than sevoflurane. Desflurane has been shown to induce a shorter duration of EA than sevoflurane [11]. Switching from sevoflurane to desflurane after the anesthetic induction may reduce the duration of EA without the risk of airway irritation. However, there has been insufficient research on the appropriate dosage of dexmedetomidine to prevent EA in children undergoing desflurane anesthesia. A sedative, dexmedetomidine, does not cause respiratory depression, but it does have cardiovascular effects, and therefore its use requires close monitoring, especially in children [12]. However, there was no definite dose of dexmedetomidine recommended to prevent EA while prolonged sedation and cardiovascular complications did not occur. The aim of this study was to identify the optimal dose of dexmedetomidine needed to prevent EA in children after a tonsillectomy or adenoidectomy under desflurane anesthesia without serious complications.

## Methods

The present study was conducted in accordance with the Declaration of Helsinki (World Medical Association). After obtaining institutional review board approval from Seoul National University Hospital and written informed consent from parents or guardians, patients eligible for inclusion in the study—those between 2 and 12 years of age, classified as ASA status I or II, and scheduled for either an adenoidectomy alone or both an adenoidectomy and tonsillectomy —were selected using Dixon’s up-and-down sequential method rather than random sequence. Patients with cardiac disease, abnormal upper airway, reactive airway diseases such as asthma, allergies to dexmedetomidine or a history of upper respiratory tract infection in the preceding 4 weeks were excluded. Patients receiving medications known to interact with dexmedetomidine such as lorazepam, diphenhydramine or furosemide were also excluded.

Intravenous accesses were secured on all children on the day before the surgery by experienced nurses. Premedication was not administered. Upon arrival in the operating room, the children were monitored with electrocardiography (ECG), pulse oximetry (SpO_2_), capnography, and noninvasive arterial blood pressure (NIBP) (Solar 8000, GE, Milwaukee, WI). In addition, the bispectral index (BIS: Vista™, Aspect Medical Systems Inc, Newton, MA) was checked. Anesthesia was induced with 6 mg/kg of sodium pentothal and 0.02 mg/kg of atropine sulfate. After loss of consciousness, children were ventilated with 8 vol. % of sevoflurane in oxygen via a pediatric circle system. They were fully relaxed with 0.6 mg/kg of rocuronium and intubated with an endotracheal tube. Anesthesia was maintained using 6–8 vol. % of desflurane in approximately 50 % oxygen in air with a total inflow of 2 L/min. The concentration of desflurane was adjusted to a BIS range of 40-60. Before the start of surgery on the first patient, 0.5 μg/kg of dexmedetomidine (Precedex inj™, 200 mcg/ml, Hospira Inc., Lake Forest, IL, USA) diluted in 10 ml of normal saline was slowly injected for 10 min and both the blood pressure and heart rate were checked at one minute intervals. Dexmedetomidine was prepared and injected by an anesthesiologist who did not participate in the assessment of EA. All tonsillectomies were performed using coblation to prevent postoperative complications including pain [13–15]. A 3 ml dose of 0.25 % bupivacaine and an epinephrine mixture (ratio of 1:200 000) were injected into the mucosa surrounding each tonsillar fossa. At the end of surgery, the desflurane was stopped and extubation was performed when the patients began breathing spontaneously and could open their eyes on command. Recovery times from the cessation of desflurane to the point at which patients’ eyes opened were recorded. The patients were transferred to the postanesthetic care unit (PACU) when fully awake. One parent was allowed to remain with the child. In the PACU, the ECG, NIBP, SpO_2,_ and the respiratory rate were checked and recorded.

EA was evaluated using the agitation scale developed by Cole *et al.* (Table 1), [16] and the worst EA score observed during the previous period was recorded by a single anesthesiologist blinded to the sequence of inclusion of patients and to the dose of dexmedetomidine administered to each patient. Recordings were made on arrival at PACU (T = 0), and 15 and 30 min later (T = 15, T = 30, respectively). EA, defined as an agitation score of 4 or 5 occurring at least once during surgery, was treated with 0.1 μg/kg of nalbuphine.

**Table 1:** Emergence agitation scale

Score	Behavior
1	Sleeping
2	Awake, calm
3	Irritable, crying
4	Inconsolable crying
5	Severe restlessness, disorientation

The dosage of dexmedetomidine was determined by Dixon’s up–and -down sequential method [17]. If a child showed EA, the next one received a dose 0.1 μg/kg greater. If no EA was observed, the next dose of dexmedetomidine was decreased by 0.1 μg/kg. The pain score was measured using the Wong-Baker face pain rating scale during the EA evaluation [18]. Nalbuphine 0.1 μg/kg was used as a rescue medicine for patients with EA or pain scores > 8. Dixon’s method requires at least six failure-success pairs for statistical analysis, therefore recruitment continued until six crossover pairs had been observed. A 50 % and 95 % effective dose of dexmedetomidine along with 95 % confidence intervals (CIs) were calculated using the isotonic regression method, [19] an adjusted response probability was obtained through use of the pooled adjacent-violators algorithm, and the CI was estimated using the bootstrapping approach [20, 21].

Demographic and recovery profile data between children with EA and those without EA were compared using Fisher’s exact test and the Mann–Whitney *U* test. Statistical analyses were performed using R − 2.15.2 for Windows and SPSS 19.0 (SPSS Inc., Chicago, IL). Statistical significance was defined as *P* < 0.05.

## Results

**Fig. 1:**
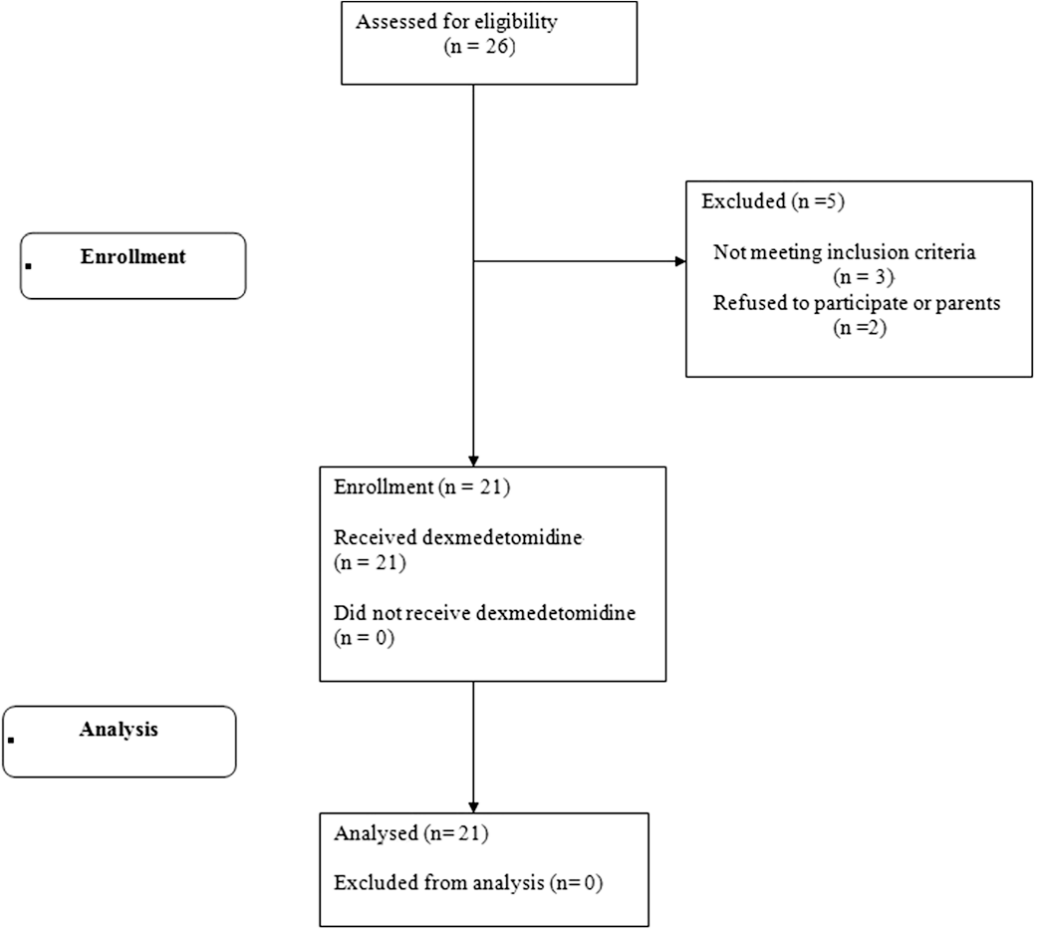
CONSORT diagram showing the flow of participants in present study

**Fig. 2:**
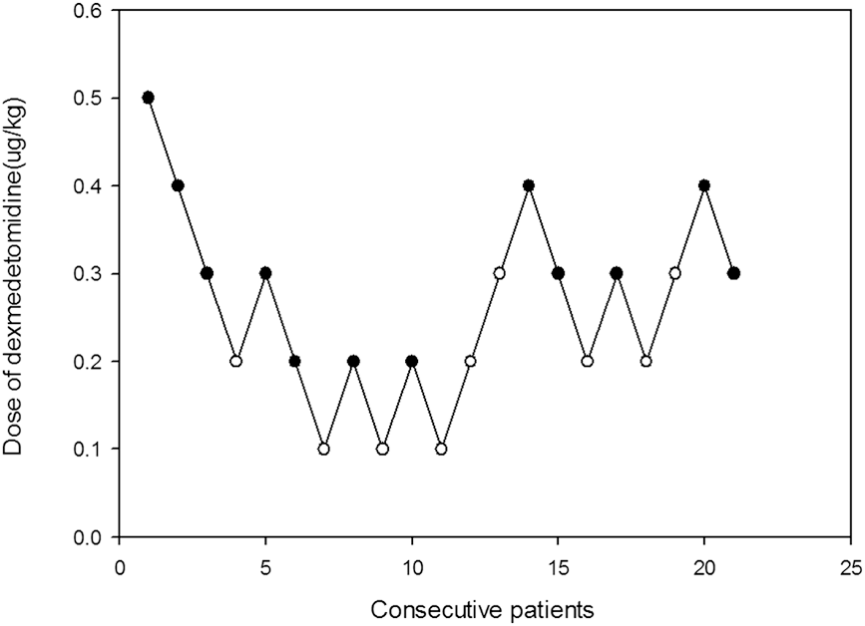
Assessment of success or failure for preventing emergence agitation (EA) in the postanesthetic care unit with dexmedetomidine by the use of Dixon’s up-and-down method. Crossover pairs from success (black circle) to failure (white circle) in preventing emergence agitation

Twenty one children were enrolled and completed this study; it is worth noting that no children dropped out, which allowed us to observe six crossover pairs (Fig. 1). Fig. 2 shows the sequences of dexmedetomidine dosages used. Neither bradycardia or hypotension occurred in any of the children following the administration of dexmedetomidine. Patient and operative characteristics were delineated in Table 2 and children with and without EA differed in demographic data or recovery profile only for the duration of their time in the PACU. The end-tidal desflurane concentration was recorded 15, 30 and 45 min after the administration of dexmedetomidine, and there was no significant difference in the mean of the end-tidal desflurane concentration between the children with and without EA. (Table 2) Nine patients developed EA in the PACU and were given nalbuphine 0.1 μg/kg, after which all had EA scores < 4. The 50 % effective dose of dexmedetomidine for the prevention of EA was 0.25 μg/kg (95 % confidence limit, 0.17–0.33 μg/kg), and the 95 % effective dose was 0.38 μg/kg (95 % confidence limit, 0.29–0.39 μg/kg). Pain scores were measured during the EA evaluation. Patients with EA had greater pain scores (median (IQR)) than those who felt relaxed (T = 0: 6.0 (5.0–9.5) vs. 4.0 (2.0–7.5), T =15: 6.0 (4.0–6.0) vs. 0 (0–2.0), and T = 30: 4.0 (3.0–5.0) vs. 2.0 (0.5–2.0), respectively). Five patients had pain scores > 8 at least once, for which they were given nalbuphine 0.1 μg/kg. The duration of patients’ PACU stay was significantly greater in those with EA than those without. No respiratory depression, hypotension or desaturation (<95 %) was observed in the PACU.

**Table 2:** Comparisons between children without EA and those with EA

	Patients without EA (n = 12)	Patients with EA (n = 9)
Male/female	3/9	2/7
Age (yr)	6.0 (3.0 – 7.0)	5.0 (3.0 – 7.0)
Weight (kg)	25.5 (20.3 – 30.5)	21.5 (15.4 – 24.9)
Height (cm)	124.3 (111.3 – 127.8)	120.0 (101.4 – 123.7)
Duration of operation (min)	36.0 (34.2 – 40.0)	31.0 (28.0 – 38.5)
Duration of anesthesia (min)	52.0 (47.2 – 60.7)	50.0 (47.0 – 57.5)
End-tidal desflurane concentration (%)	6.1 ± 0.9	6.3 ± 1.0
Recovery time from anesthesia (min)	8.0 (7.0 – 9.0)	8.0 (6.25 – 10.0)
PACU Stay (min)	32.0 (27.0 – 34.0)	39.0 (34.0 – 42.5)^*^

EA, emergence agitation

Values are presented as number of patients, or the median (interquartile range)

Age is presented as the median (range)

*P < 0.01

## Discussion

Inhaled anesthetic agents with low blood-gas solubility such as sevoflurane and desflurane have clinical advantages in allowing rapid induction and emergence. Sevoflurane is widely used as an inhalation anesthetic in children to avoid the airway irritation that desflurane causes during the anesthetic induction period. Sevoflurane anesthesia, however, is associated with a higher incidence of EA in children when compared with other volatile anesthetics or propofol whereas [22–25] desflurane is also associated with EA in children with a similar incidence but shorter duration of EA [11, 26, 27]. Desflurane may have the clinical advantage of reducing the duration of EA in children, therefore, there was a need to evaluate the optimal dose of dexmedetomidine to prevent EA in children under desflurane anesthesia. In our study of children undergoing either a tonsillectomy or adenoidectomy under desflurane anesthesia, 0.25 μg/kg of dexmedetomidine prevented EA in 50 % of children, and 0.38 μg/kg prevented EA in 95 %.

For the prevention of EA in children, various doses of a single bolus of dexmedetomidine (0.15–1.0 μg/kg) or timings of the single bolus of dexmedetomidine (immediately after anesthesia induction or 5 min before the end of surgery) have been recommended [5, 7, 28]. Continuous infusion also decreases the incidence and frequency of EA in children with sevoflurane anesthesia [9, 29]. Although these previous studies focused on whether dexmedetomidine reduces the incidence of EA in children, there has been little research into what dosage of dexmedetomidine induces prolonged sedation or cardiovascular instability. The 50 % and 95 % effective doses of dexmedetomidine for the prevention of EA (0.25 μg/kg and 0.38 μg/kg, respectively) determined using Dixon’s up–and-down sequential method in the present study are relatively low when compared to those reported in previous studies. These effective doses can serve as appropriate guidelines for not only preventing EA but also reducing the risk of complications related to dexmedetomidine overdose.

Even with the relatively high doses of dexmedetomidine (0.4 μg/kg or 0.5 μg/kg) used in this study, we did not observe complications of dexmedetomidine such as bradycardia, hypotension or prolonged recovery time. The reason may be that the doses of dexmedetomidine used in the present study were not high enough to induce these complications [30]. The timing of dexmedetomidine administration may also have prevented complications. A bolus of dexmedetomidine was given immediately after anesthesia induction. Stimulus by tracheal intubation and 0.02 mg/kg of atropine sulfate given before anesthesia induction might also prevent such hemodynamic changes. Considering the short half-life (2 h) of dexmedetomidine, it can be metabolized after being administered, thus preventing a prolonged recovery time.

There are several agitation scales, such as the Pediatric Anesthesia Emergence Delirium, which are used to evaluate EA in children. The present study used an agitation scale that was employed in previous studies reporting a preventive effect of dexmedetomidine on EA in children [7–9]. The dose of dexmedetomidine (0.5 mcg/kg) administered to the first child in our study was based on the results of these previous studies. We hypothesized that using the same scale to evaluate EA would be appropriate for determining the effective dose of dexmedetomidine to prevent it and for comparison to previous studies. However, a single EA scale was used to define it in the present study. The evaluation of EA could have been more reliable if an additional EA scale had been used in conjunction. After all, caution should be exercised when applying the results of the present study to patients while using different scales and definitions of EA for the assessment of EA.

Postoperative pain was more severe in patients with EA than in those without EA in the present study. This is for two reasons: first, postoperative pain can be a cause of EA, and secondly, the EA scale and Wong-Baker face pain rating scale have several common features. The EA evaluation should have been conducted when children were in a pain-free state to rule out the effect of postoperative pain. However, the aim of the study was to determine the effective dose of dexmedetomidine necessary to prevent EA in children undergoing tonsillectomies or adenoidectomies, and several methods, were performed to reduce postoperative pain, such as a surgical technique which uses coblation and the mucosal injection of local anesthetics surrounding the tonsillar fossa. In addition, dexmedetomidine has been reported to reduce postoperative opioid requirements after a tonsillectomy and adenoidectomy [29]. As a result, despite the absence of an analgesic administration before emergence, the incidence of postoperative pain was lower in patients the present study than in those who had received acetaminophen in previous studies [7].

This study had certain limitations which make it difficult to draw comparisons to previous studies. First, EA can be influenced by many factors (pain, hunger, post-operative nausea and vomiting, and fear of strangers) during the perioperative period. Furthermore, though Dixon’s up-and-down sequential method was used, the sample size of the present study was relatively small. Further clinical research to validate the suggested dose of dexmedetomidine to prevent EA should be performed in the future. Second, the ages of the children in the present study were intended to range from 2 to 12 years old. However, in practice, the age range of the children enrolled was 3–7 years old, and the influence of age on the dose of dexmedetomidine may be not significant. Finally, children in the present study did not receive any premedication so as to avoid additional variability of responses. The effective dose of dexmedetomidine to prevent EA may have been different if children had received premedication to relieve their anxiety before the anesthetic induction.

## Conclusions

In conclusion, to prevent EA in 50 % or 95 % of children undergoing tonsillectomies or adenoidectomies following desflurane anesthesia, a single bolus of 0.25 or 0.38 μg/kg of dexmedetomidine is suggested. Further clinical trials need to be conducted in order to validate the suggested dose of dexmedetomidine to prevent the incidence of EA that was identified in the present study.

## Abbreviations

EA: Emergence agitation
ECG: Electrocardiography
SpO_2_: Pulse oximetry
NIBP: Noninvasive arterial blood pressure
BIS: Bispectral index
PACU: Postanesthetic care unit
CIs: Confidence intervals.
